# Rapid Evaporative Ionisation Mass Spectrometry (REIMS) Provides Accurate Direct from Culture Species Identification within the Genus *Candida*

**DOI:** 10.1038/srep36788

**Published:** 2016-11-14

**Authors:** Simon J. S. Cameron, Frances Bolt, Alvaro Perdones-Montero, Tony Rickards, Kate Hardiman, Alireza Abdolrasouli, Adam Burke, Zsolt Bodai, Tamas Karancsi, Daniel Simon, Richard Schaffer, Monica Rebec, Julia Balog, Zoltan Takáts

**Affiliations:** 1 Section of Computational and Systems Medicine, Department of Surgery and Cancer, Imperial College London, London, SW7 2AZ, United Kingdom; 2 Department of Microbiology, Imperial College Healthcare NHS Trust, Charing Cross Hospital, London, W6 8RF, United Kingdom; 3 Fungal Pathogens Immunobiology Laboratory, National Heart and Lung Institute, Imperial College London, SW7 2AZ, United Kingdom; 4 Waters Research Centre, 7 Zahony Street, Budapest, 1031, Hungary.

## Abstract

Members of the genus *Candida*, such as *C. albicans* and *C. parapsilosis*, are important human pathogens. Other members of this genus, previously believed to carry minimal disease risk, are increasingly recognised as important human pathogens, particularly because of variations in susceptibilities to widely used anti-fungal agents. Thus, rapid and accurate identification of clinical *Candida* isolates is fundamental in ensuring timely and effective treatments are delivered. Rapid Evaporative Ionisation Mass Spectrometry (REIMS) has previously been shown to provide a high-throughput platform for the rapid and accurate identification of bacterial and fungal isolates. In comparison to commercially available matrix assisted laser desorption ionisation time of flight mass spectrometry (MALDI-ToF), REIMS based methods require no preparative steps nor time-consuming cell extractions. Here, we report on the ability of REIMS-based analysis to rapidly and accurately identify 153 clinical *Candida* isolates to species level. Both handheld bipolar REIMS and high-throughput REIMS platforms showed high levels of species classification accuracy, with 96% and 100% of isolates classified correctly to species level respectively. In addition, significantly different (FDR corrected *P* value < 0.05) lipids within the 600 to 1000 *m/z* mass range were identified, which could act as species-specific biomarkers in complex microbial communities.

The rapid diagnosis of fungal infections is critical in ensuring that appropriate and timely antifungals can be administered^1^. This, in turn, will improve patient outcomes and reduce both costs and the unnecessary use of potentially toxic antifungals. Here, we describe the utilization of REIMS technology for the taxonomical classification of *Candida* species. Whilst members of the genus *Candida* are found within the environment, and are a common constituents of the human microbiota, they can cause severe disease, particularly in immunocompromised patients^2^,^3^. The organism is associated with a wide spectrum of disease from cutaneous and mucosal candidiasis to systemic infections which have high mortality rates ranging from 15 to 35%^4^. Although *Candida albicans* is the most common *Candida* species, non-*albicans* species including *C. glabrata*, *C. tropicalis*, *C. krusei* and *C. parasilopsis* are increasingly being isolated and now account for 35–65% of invasive candidiasis cases^5^. Identifying the causative species is critical for effective clinical management as many species have intrinsic resistance to antifungal classes. For example, whilst *C. albicans* is typically susceptible to azoles, *C. krusei* and *C. inconspicua* are innately resistant^5^. Furthermore, species specific antifungal interpretative breakpoints have been introduced; making it a necessity to accurately speciate *Candida* isolates^6^,^7^,^8^,^9^.

Rapid Evaporative Ionisation Mass Spectrometry (REIMS) has previously been shown to successfully characterise and identify clinically significant bacteria and yeasts^10^,^11^. Whilst early studies utilised bipolar forceps, we have recently developed a high throughput adaptation, suitable for clinical laboratories. This platform incorporates an adapted liquid handling instrument, modified to allow analysis of microbial colonies with monopolar probe, with an integrated plate and colony visualisation platform for automated colony picking. Species specific mass spectral fingerprints are generated by applying a radiofrequency electrical current directly to the microbial colony using a stainless steel monopolar probe. The resulting vapour, containing gas phase ions of metabolites and structural lipids, is channelled to mass spectrometer using the instrument’s vacuum system; allowing for mass spectral data to be generated within one second of sample heating^12^.

Traditionally, the identification of members of the genus *Candida* within a clinical laboratory relied upon biochemical assays and the germ tube test^9^,^13^. Molecular methods including sequencing of the 18S rRNA gene and Internal Transcribed Spacer (ITS) regions can be used in instances where species delineations are poor. However, these methods are time consuming, costly and many clinical laboratories do not have access to appropriate facilities^14^. However, the introduction of mass spectrometry into clinical microbiology laboratories has revolutionised fungal diagnostics^15^. Commercially available mass spectrometry platforms utilise matrix assisted laser desorption ionisation (MALDI-ToF) mass spectrometry. These platforms compare mass spectral fingerprints of predominately ribosomal proteins to a database to assign genus and/or species classifications^16^. For bacterial isolates, an isolated colony is spotted onto a stainless steel plate and overlaid with a matrix to facilitate ionisation. However, for the analysis of fungal isolates, a lengthy extraction procedure involving multiple solvent extraction and centrifugations steps is required to release intracellular proteins before the matrix can be applied and analysis can be undertaken^17^,^18^,^19^. This laborious method increases costs, user input and the time to identification. In turn this limits the number of yeasts that can be routinely identified using a MS approach.

Here, we report on the use of high-throughput REIMS analysis to assign species level taxonomic classification to 153 clinical *Candida* isolates with perfect accuracy. In contrast to commercially available MS methods, REIMS analysis is performed directly from a culture plate, with no requirement for sample pre-treatment or extraction, and thus allows for a substantially higher throughput than MALDI-ToF platforms.

## Results and Discussion

A total of 161 *Candida* isolates were collected and subjected to species level identification using both MALDI-ToF mass spectrometry and DNA sequencing of the ITS region. Using these two approaches, eight *Candida* isolates gave conflicting species level identifications, Supplementary Table 1. Merged sequence reads and ITS database match statistics are given in Supplementary Data Matrix 1. To ensure that a true reflection of the capacity of REIMS to perform species level classification within the genus *Candida*, these eight isolates were removed from subsequent analysis. This resulted in a total of 153 *Candida* isolates with dual confirmation of species level identification.

**Figure 1:**
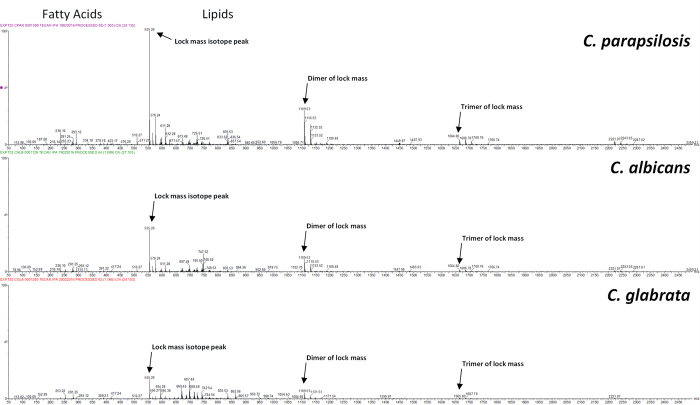
Representative Mass Spectra from *Candida* Species. Representative mass spectra, after background subtraction, mass drift correction, and removal of lock mass compound peak (≈554 *m/z*), of *C. parapsilosis*, *C. albicans*, and *C. glabrata* are shown. Major mass spectral features are labelled, such as the fatty acid and lipid mass regions, and also the isomer, dimer, and trimer peaks of the lock mass compound used for mass drift correction. For all three *Candida* species shown, peaks within the lipid region (600 to 1000 *m/z*) show the highest signal intensity.

Representative spectra of three *Candida* species are shown for the high-throughput REIMS platform, Figure 1, which show high signal intensity is visible within the mass regions containing fatty acids (≈50 to ≈500 *m/z*) and lipids (≈600 to ≈1000 *m/z*). Mean mass spectra for each of the eight *Candida* species analysed, Supplementary Figures S1 to S8, show that similar spectral fingerprints are obtained for each species using both handheld bipolar REIMS and high-throughput REIMS. There are however, differences between *Candida* species, with substantial variations in peak intensities of those occurring below 600 *m/z*, and also of major peaks within the 600 to 1000 *m/z* range.

**Figure 2:**
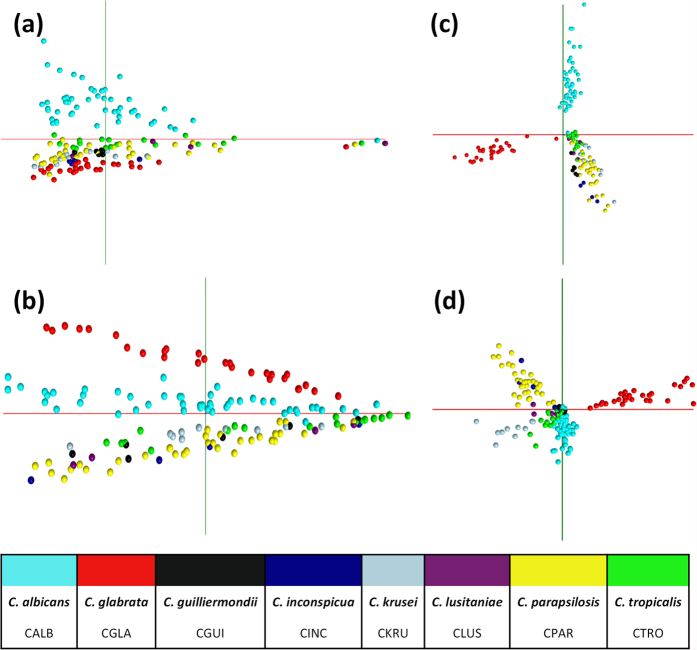
Multivariate Modelling of Entire Mass Spectral Acquisition. Principal component analysis of the entire mass spectral acquisition range (50 to 2500 *m/z*) was completed using OMB software for (**a**) handheld bipolar REIMS and (**b**) the high-throughput REIMS platform. In both, separation of *C. albicans* isolates is evident, with greater separation observed for high-throughput REIMS. Supervised linear discriminant analysis plots, using seven PCA dimensions, shows greater separation of both *C. alibicans* and *C. glabrata* isolates in both (**c**) handheld bipolar REIMS and (**d**) high-throughput REIMS, with overlapping clusters of other *Candida* isolates.

Initial multivariate analysis of the experimental acquisition (50 to 2500 *m/z*) range, Figure 2, shows separation of *C. albicans* isolates in principal component analysis of both handheld bipolar REIMS, Figure 2a, and high-throughput REIMS, Figure 2b; with the latter also showing separation of *C. glabrata* isolates. Supervised multivariate analysis using linear discriminant analysis shows improved separation in isolates analysed using both REIMS approaches, Figure 2c,d. This improvement appears to be strongest within *Candida* species which showed good separation using principal component analysis modelling. Substantial overlap however, is evident between other *Candida* species; suggesting that using the entire experimental acquisition range of the Xevo G2-XS Q-ToF instrument is not optimum for species classification. Indeed, initial Random Forest species classification using this mass range, a mass bin of 0.1 Da, and 400 decision making trees gave 91% and 93% accuracy using handheld bipolar REIMS and high-throughput REIMS respectively.

### Effect of Restricted Mass Range on Species Classification Accuracy

In order to identify whether using a restricted mass range improved species classification accuracy, a Random Forest feature selection tool was used to identify the 250 most important mass bins, at 0.1 Da, for classification across the 50 to 2500 *m/z* range. Supplementary Figure S9 shows that for both REIMS approaches, the highest number of important mass bins reside within the 600 to 1000 *m/z* range. Although other mass ranges contain important features, the sum of their relative importance to the Random Forest classification model’s accuracy is lower than for the 600 to 1000 *m/z* range. This further suggests that this restricted mass range may allow for improved classification accuracy of Random Forest models.

**Figure 3:**
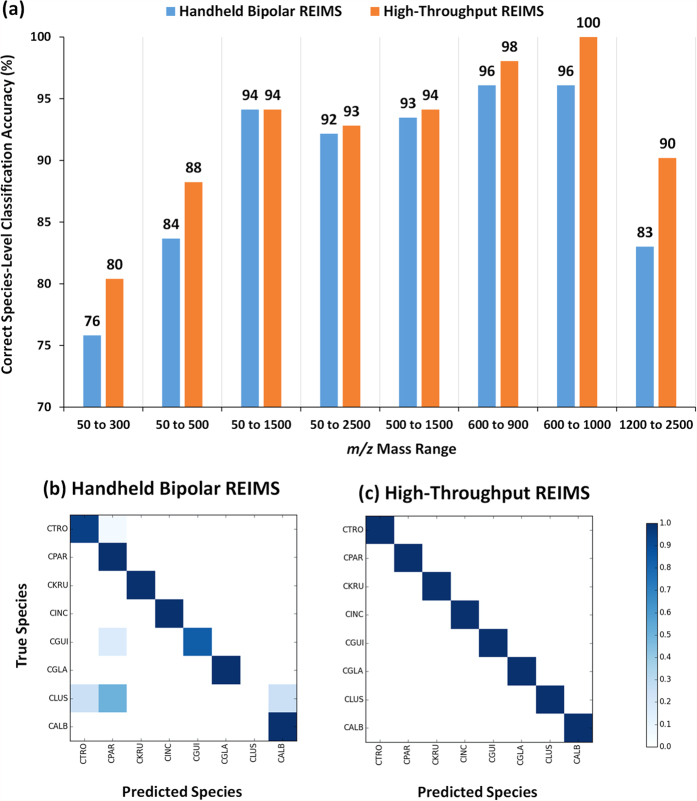
Species Classification Accuracy using Restricted Mass Ranges. The species classification accuracy of (**a**) seven restricted mass ranges were compared. For all restricted mass ranges, the species classification accuracy of high-throughput REIMS out-performed handheld bipolar REIMS. For both REIMS approaches, the restricted mass range of 600 to 1000 *m/z* provided the highest species classification accuracy. Species classification confusion matrices are shown for (**b**) handheld bipolar REIMS, and (**c**) high-throughput REIMS for this restricted mass range. For each colour square, the darkness of blue indicates the level of species classification accuracy, with the darkest colour representing 100% accuracy. Random Forest models constructed on given mass ranges with a mass bin of 0.1 Da and using 400 decision making trees.

To assess this, Random Forest models of seven restricted mass ranges: 50 to 300 *m/z*, 50 to 500 *m/z*, 50 to 1500 *m/z*, 500 to 1500 *m/z*, 600 to 900 *m/z*, 600 to 1000 *m/*z, and 1200 to 2500 *m/z* were created and their classification accuracy compared to that of the 50 to 2500 *m/z* mass range. Leave one out cross-validation of Random Forest models, Figure 3a, showed that for both handheld bipolar REIMS and high-throughput REIMS, the 600 to 1000 *m/z* restricted mass range provided the highest level of species classification accuracy, with 96% and 100% accuracy achieved respectively. This mass range predominately contains ionised lipids^20^,^21^,^22^, the utilisation of which have a long history in bacterial classification^23^,^24^,^25^. Additionally, they have also been used for the classification of yeasts, including species of the *Candida* genus^26^. In addition to providing the highest level of species classification accuracy, utilisation of the restricted mass range of 600 to 1000 *m/z* also reduces computational requirements substantially, allowing for rapid species classification.

To tentatively identify the mass spectral features important to Random Forest model classifications, the Random Forest feature selection tool was used to identify ten mass bins, at 0.1 Da, with the highest importance values in models for both REIMS approaches. The LIPID MAPS database was then used to assign tentative lipid identifications to these mass bins, after interrogation of the normalised mass spectra to identify the two decimal place mass of the major peak near each 0.1 Da bin. Supplementary Table S3 gives the tentative lipid identifications of the ten important mass bins contributing to the Random Forest classification for both REIMS approaches. A number of these lipids have been previously identified in *Candida* species and interestingly, have been shown to be statistically different in abundance between different species within the genus^27^,^28^.

### Identification of Optimal Mass Bin for Accuracy of Species Classification

After identification of the 600 to 1000 *m/z* range as optimal for classification accuracy, the effect of mass bin size was also assessed. Within this restricted mass range, the species classification accuracy of Random Forest models using mass bins of 0.01 Da, 0.05 Da, 0.1 Da, 0.5 Da, and 1 Da sizes were calculated. When comparing both REIMS approaches (shown in Figure 3), no consistent pattern of mass bin size dependence was evident. For handheld bipolar REIMS, the highest classification accuracy was achieved with all mass bins except 0.5 Da. For high-throughput REIMS however, the mass bin of 0.1 Da achieved the highest, 100%, species level classification. At all levels of mass bin size, high-throughput REIMS outperformed handheld bipolar REIMS with regard to species level classification accuracy.

To achieve an optimum balance between species level classification accuracy and computational requirements, a mass bin of 0.1 Da was used for all subsequent data analysis. Visual inspection of mass spectra, after background removal and mass drift correction, during peak identification suggested that there was minimal sharing of important mass bins between two or more mass peaks. This gives a possible explanation for the apparent lack of a substantial mass bin size effect in classification accuracy; as an effect would only be expected if increasing the number of decimal places for mass bins allowed for two or more important spectral features to be separated.

### Optimisation of Decision Making Tree Number in Random Forest Analysis

The number of decision making trees constructed in each Random Forest can be user-defined to any integer value. In species classification models described thus far, a total of 400 decision making trees, an arbitrarily chosen number, have been constructed in each model. Random Forest models can reach a plateau in regards to classification performance; whilst computational requirements continue to increase substantially^29^. To determine the number of decision making trees required to reach either a maximal level of species level classification accuracy, or a plateau, Random Forest models were constructed using 10 to 2000 trees, with interval increases as shown in Supplementary Figure S11. For both REIMS approaches, a plateau was reached after the maximum classification accuracy had been achieved. For handheld bipolar REIMS, the highest level of classification accuracy was achieved whilst using 100 decision making trees, and for high-throughput REIMS, whilst 400 decision making trees were used. So that the same number of decision making trees were employed in Random Forest models for both REIMS approaches, 400 trees were used in further analysis for individual species level classification accuracy. Although, this number is optimised with regards to the high-throughput platform, it should be noted that at all numbers of decision making trees used in Random Forest species classification, this REIMS approach outperformed the handheld bipolar REIMS approach by at least two percentage points.

### Comparison of Species Classification Accuracy between Handheld and High-Throughput REIMS

Following optimisation of the Random Forest model parameters the classification accuracy for each of the eight *Candida* species was determined. Confusion matrices for handheld bipolar REIMS, Figure 3b, and high-throughput REIMS, Figure 3c, reinforce that the latter consistently gives higher levels of species classification accuracy within the *Candida* genus. Precision, sensitivity, and F1 scores (harmonic mean of precision and sensitivity scores) for each *Candida* species, are given in Table 1, for both REIMS approaches. For handheld bipolar REIMS, misclassifications occurred for isolates within three species: *C. tropicalis*, *C. guilliermondii*, and *C. lusitaniae*; whilst no instances of miss-classifications were observed for high-throughput REIMS.

Here, high-throughput REIMS provides greater species classification accuracy within the *Candida* genus than handheld bipolar REIMS. The fungal cell wall is compositionally different to bacterial cell envelopes, with the former possessing high amounts of chitin, glucans, and glycoproteins^30^. It may be that the higher temperature generated at the point-of-contact between the stainless steel monopolar probe and microbial biomass in high-throughput REIMS is necessary to maximise the ionisation of structural lipids, thereby improving taxonomical classification accuracies.

### Identification of Species Biomarkers through Univariate Statistics

**Figure 4:**
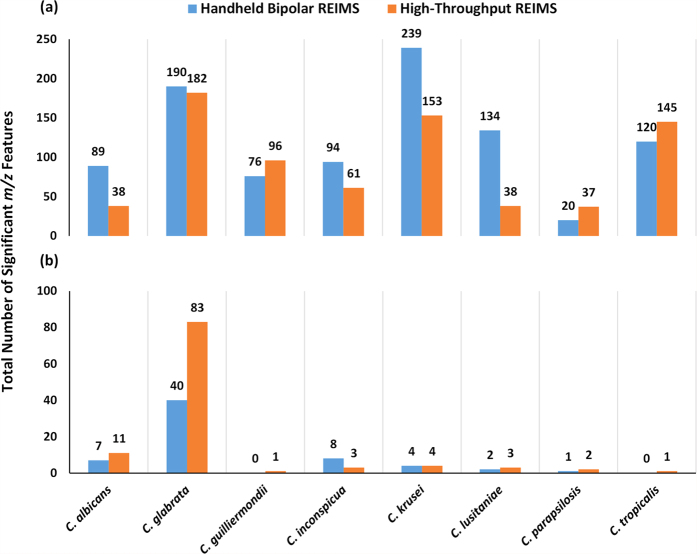
Significant Mass Spectral Features Identified through Univariate Analysis. Univariate analysis of variance statistics were calculated for all mass spectral features within the 600 to 1000 *m/z mass* range, at a mass bin of 0.1 Da. Only those mass spectral features with an FDR corrected *P* value below 0.05 were used in subsequent calculations. The total number of mass spectral features which are (**a**) higher in each *Candida* species, compared to all seven others, and the total number which are (**b**) lower in each *Candida* species, compared to all seven others, are shown.

**Figure 5:**
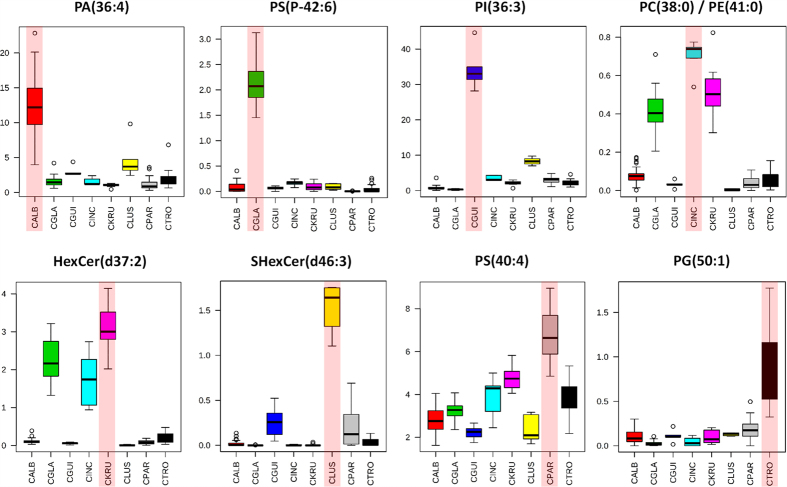
Box Plots of Top Ranked Significant Mass Spectral Features for High-Throughput REIMS. Box plots for the highest ranked mass spectral bin, with tentative compound identification, by FDR corrected *P* value, for each of the eight *Candida* species analysed, identified using high-throughput REIMS analysis. All eight *Candida* species are separated by higher intensity levels of mass spectral feature. Red shaded column indicates *Candida* species separated by mass spectral feature. Full species name for each abbreviation is given in Table 1.

Thus far, we have reported on supervised multivariate statistical analysis to determine the species level accuracy of Random Forest models. Although we have substantially reduced the computational requirements of this approach through optimisation of our data analysis parameters, alternative methods of data analysis may allow for further reductions to be achieved. Mass spectral features that significantly differ between *Candida* species were identified using a univariate analysis of variance approach. This univariate analysis revealed a substantial number of significant mass spectral bins which separate *Candida* species, as shown by Figure 4. These were either significantly higher in individual *Candida* species, Figure 4a, or significantly lower, Figure 4b. The top ranked mass spectral feature, by FDR corrected *P* value, for high-throughput REIMS is displayed in Figure 5. Tentative compound identification was completed, in the same way as for important Random Forest model features, for the highest ranking significant feature, Supplementary Table S3. As before, a number of these tentatively identified lipid compounds have previously been reported as significantly different between *Candida* species^27^.

In addition to providing structure to the cellular membrane, lipids have been associated with differences in antifungal sensitivities between *Candida* species. For example, variations in the phosphatidylcholine to phosphatidylethanolamine ratio of *C. albicans* plasma membranes has been associated with azole resistance^31^. Additionally, *C. lusitaniae* isolates are frequently reported as being resistant to amphotericin B, which is often associated with changes to the lipid composition of plasma membranes, and particularly a reduction in ergosterol^32^. *Candida* lipids have also been associated with virulence factors, namely adherence to human buccal epithelial cells; which is an essential step in host infection^33^. Therefore, REIMS may provide both a platform for direct-from-culture identification of *Candida* isolates, and also identification of antifungal and virulence phenotypes.

## Concluding Remarks

Here, we have shown that our recently reported high-throughput REIMS platform is able to assign taxonomic classifications to *Candida* isolates with near perfect species level accuracy. Unlike current, commercially available platforms, REIMS can be used to analyse *Candida* cultures directly from culture; without a laborious and time-consuming extraction protocol. High-throughput REIMS requires minimal user input, can easily be incorporated into the workflow of automated clinical microbiology laboratories. Although our statistical analysis has focussed on the restricted mass range of 600 to 1000 *m/z*, the fungal metabolites identified within this range, and also outside of this acquisition range may have further utility in the characterisation of clinically important phenotypes; such as antifungal resistance mechanism and virulence.

## Experimental Section

### Culturing of *Candida* Isolates

A total of 161 isolates, from eight *Candida* species, detailed in Supplementary Table S1, were collected as part of routine clinical work at Imperial College Healthcare NHS Trust’s (London, UK) Microbiology Diagnostic laboratory. Each *Candida* isolate was grown under the culture conditions detailed in Supplementary Table S1 prior to REIMS analysis.

### Species Identification of *Candida* Isolates using MALDI-ToF Mass Spectrometry

Species level identification of *Candida* isolates was initially completed using matrix assisted laser desorption ionisation time of flight (MALDI-ToF) mass spectrometry on the Bruker Microflex LT (Bruker Daltonics, Coventry, UK) system, following the manufacturer’s standard instructions. For each *Candida* isolate, a single colony was suspended in 1 mL of 70% ethanol (*V*/*V* with water) and mixed until homogenous. After the sample underwent centrifugation at 15,000 × *g* for 2 minutes, the supernatant was removed and the remaining pellet dried at room temperature for 30 minutes. After drying, between 10 μL and 60 μL (depending on size of cell pellet) of 70% formic acid (*V*/*V* with water) was added and the pellet suspended through pipetting. An equal amount of pure acetonitrile was then added, in relation to the previously added 70% formic acid (*V*/*V* with water), and incubated at room temperature for 3 minutes. The sample then underwent centrifugation at 15,000 × *g* for 3 minutes with 1 μL of the supernatant then spotted onto an analysis plate. After the spotted supernatant had dried at room temperature, it was overlaid with 1 μL of α-cyano-4-hydroxycinnamic acid (HCCA) matrix, (Bruker Daltonics) dissolved in 50% acetonitrile, 47.5% water, and 2.5% trifluoroacetic acid. The matrix was allowed to dry at room temperature before analysis following the manufacturer’s instructions. Isolates were analysed using the Bruker Biotyper software (version 3.0) and library (version 5.0) for taxonomic classification.

### Species Identification of *Candida* Isolates using DNA Sequencing of ITS Region

*Candida* isolates were subjected to genotypic identification through sequencing of the internal transcribed spacer (ITS) region. DNA was extracted from *Candida* isolates, after culturing as previously described, through the suspension of one culture colony into 200 μL of Prepman Ultra reagent (Life Technologies, California, USA), also containing 100 mg of 0.5 mm silica/zirconia beads (Thistle Scientific, Glasgow, UK). The suspension was then subjected to vortex mixing for 30 seconds and then heated at 100 °C for 15 minutes in a ThermoMixer C instrument (Eppendorf, Stevenage, UK) set at 500 rpm. Suspensions were then subjected to bead beating in a FastPrep-24 5G instrument (MP Biomedicals, UK) for 30 seconds at a speed setting of six. After cooling at −80 °C for two minutes, suspensions were subjected to heating at 100 °C for ten minutes as previously described. Suspensions then underwent centrifugation at 21,000 × *g* for two minutes to pellet cell debris and 100 μL of supernatant removed; of which 5 μL was diluted with 45 μL of sterile molecular grade water prior to use in polymerase chain reactions (PCR). PCR was conducted in 50 μL reaction volumes consisting of 3 μL of template DNA, 25 μL of 2X BioMix polymerase (BioLine, UK), 3 μL each of ITS-1 (5′-TCC GTA GGT GAA CCT GCG G -3′) and NL-4 (5′-GGT CCG TGT TTC AAG ACG G -3′) primers at stock concentrations of 10 μM, and 16 μL of PCR grade water (Roche, UK). PCR reaction volumes were then subjected to heating at 95 °C for five minutes, 35 cycles consisting of heating at 94 °C for 30 seconds, 60 °C for 30 seconds, and 72 °C for 60 seconds, followed by a final elongation step for ten minutes at 72 °C. PCR products were verified through visualisation on a 1.0% agarose gel, after separation at 110 mA for 60 minutes, stained with SYBR Safe DNA Gel Stain (ThermoFisher Scientific, UK), which was imaged using an Azure c200 system. PCR products were sequenced using an ABI 3730 XL system using ITS-1 (5′-TCC GTA GGT GAA CCT GCG G -3′) and ITS-4 (5′-TCC TCC GCT TAT TGA TAT GC -3′) primers by LGC Genomics (Berlin, Germany). Resulting paired sequence data was merged using the SeqTrace^34^ (version 0.9) programme under standard parameters and a minimum confidence Bayesian score of 15. Merged reads were then used to interrogate the International Society of Human and Mycology (ISHAM)-ITS reference DNA barcoding database^35^. Species level identifications were recorded when the ITS sequence used to interrogate the database returned a match with a sequence similarity score greater than 97%, and a coverage in excess of 97%. Where more than one result was returned, the entry with the highest similarity score was used for species level identification of the isolate.

### Handheld Bipolar and High-Throughput REIMS Analysis of *Candida* Isolates

Analysis of *Candida* isolates was conducted using both the handheld bipolar and high-throughput REIMS approaches. For the former, a handheld bipolar probe device (Erbe Elektromedizin, Tübingen, Germany) was combined with an Erbe ICC 300 electrosurgical generator (Erbe Elektromedizin) operated at a power setting of 70 W in bipolar mode with a radiofrequency alternating current power supply (470 kHz sinusoid). The handheld bipolar probe was connected to the Xevo G2-XS Q-ToF mass spectrometer’s inlet capillary via a *ca*. 1.5 m long (inner diameter 1.5 mm, outer diameter 3.2 mm) PTFE tube (Sigma-Aldrich, UK) to allow transfer of the generated analyte vapour. For each *Candida* isolate, a microbial biomass between 0.1 and 1.5 mg was removed from each culture plate using one side of the probe device, which was then closed and the power supply triggered using a manual foot switch. High throughput analysis was performed using a modified colony picker robot (Freedom EVO platform (TECAN, Switzerland)) linked with a plate visualisation and colony picker platform (SciRobotics, Israel). Bespoke stainless steel electrode probes, modified from a 200 μL pure tip (TECAN) were used in combination with the previously detailed electrosurgical generator, set at a heating power of 17 W in monopolar mode. For both REIMS analysis methods, the analyte vapour was co-aspirated with isopropanol, flow rate of 0.2 mL/min, containing leucine-enkephalin at a concentration of 10 ng/μL. For each *Candida* isolate, a total of five individual measurements were performed. Raw spectral data files are available via the MetaboLights online repository under study identifier MTBLS383.

### Mass Spectrometry Analysis using Xevo G2-XS Q-ToF Instrument

Mass spectrometry analysis of the REIMS analyte vapour was completed using a Xevo G2-XS Q-ToF (Waters Corporation, Wilmslow, UK), with the instrument settings as detailed in Supplementary Table S2. The Xevo G2-XS Q-ToF instrument was calibrated daily using sodium formate in negative ionisation mode, following the manufacturer’s standard instructions. All mass spectrometry analysis was performed in negative ionisation mode with mass spectra acquired over the 50 to 2500 *m/z* range.

### Data Analysis

After mass spectrometry acquisition, all raw data files were imported into the Offline Model Builder (version 1.1.29.0) software (Waters Research Centre, Budapest, Hungary) for pre-processing. Initially, each scan was combined into spectra, resulting in five individual replicate spectra for each *Candida* isolate. The spectra were then subjected to a background subtraction algorithm, and lockmass corrected to the external compound leucine-enkephalin (negative ionisation *m/z* 554.2615) contained within the isopropyl alcohol matrix. Spectra were then subjected to total ion count normalisation and re-binned to a user-defined Da value for further data analysis. For Random Forest analysis^29^, a mass spectral matrix after data pre-processing in the Offline Model Builder Software was imported into the machine learning scikit-learn package^36^, via a bespoke graphical user interface. This interface utilised Cytoscape.js^37^ and plotly.js packages for data visualisation. This package was used for both sample classification and identification of important mass spectral bins used in sample classification models. The number of decision making trees was defined by the user, and the Random Forest algorithm expanded all trees until the ‘leaves’ were considered pure. Leave one out cross-validation was completed to determine the classification accuracy of each Random Forest model. Univariate data analysis was completed using the MetaboAnalyst 3.0^38^ online platform. Analysis of variance was performed on 0.1 Da binned data encompassing the 600–1000 *m/z* range which was exported as a pre-processed data matrix from the Offline Model Builder software. Fisher’s least significant difference test was used by the MetaboAnalyst 3.0 platform as a *post hoc* test to identify significantly different *Candida* species. Only those mass spectral features with false discovery rate corrected *P* values below 0.05 were used to calculate the number of significant features for each *Candida* species.

### Tentative Identification of Mass Spectral Features

Using both Random Forest classification and univariate ANOVA analysis, important and significant mass bins were identified within the 600 to 1000 *m/z* range. To assign tentative identifications to these mass spectral features, spectra, after background removal and mass drift correction using MassLynx (version 4.1) software (Waters Corporation), were interrogated to identify peaks within these mass bins. These mass spectral peak, using a mass accuracy of two decimal places, were used to interrogate the virtual database of lipids within the LIPID MAPS^39^ database; allowing for a mass tolerance of +/−0.5 *m/z*, and negative ion types. The highest ranking database match, based on Delta score value, was used for the tentative identification of interrogating mass spectral peak. Where the highest Delta score value was shared by more than one lipid class, both entries were given as tentative identifications.

### Safety Considerations

Throughout the work detailed here, all *Candida* isolates were treated as potential Hazard Group 2 organisms and thus, were handled at all times within containment level two facilities. All REIMS analysis was completed within a class 2 biological safety cabinet. All chemicals and solvents used in this work were handled according to their material safety data sheet provided by the relevant manufacturer.

## Additional Information

**How to cite this article**: Cameron, S. J. S. *et al*. Rapid Evaporative Ionisation Mass Spectrometry (REIMS) Provides Accurate Direct from Culture Species Identification within the Genus *Candida*. *Sci. Rep*. **6**, 36788; doi: 10.1038/srep36788 (2016).

**Publisher’s note:** Springer Nature remains neutral with regard to jurisdictional claims in published maps and institutional affiliations.

## Supplementary Material

Supplementary Information

Supplementary Data S1

## Figures and Tables

**Table 1:** Precision, Sensitivity, and F1 Scores from Random Forest Species Classification.

	Handheld Bipolar REIMS			High-Throughput REIMS		
	Precision (%)	Sensitivity (%)	F1 Score (%)	Precision (%)	Sensitivity (%)	F1 Score (%)
*C. albicans* (CALB)	98	100	99	100	100	100
*C. glabrata* (CGLA)	100	100	100	100	100	100
*C. guilliermondii* (CGUI)	100	83	91	100	100	100
*C. inconspicua* (CINC)	100	100	100	100	100	100
*C. krusei* (CKRU)	100	100	100	100	100	100
*C. lusitaniae* (CLUS)	0	0	0	100	100	100
*C. parapsilosis* (CPAR)	89	100	94	100	100	100
*C. tropicalis* (CTRO)	94	94	94	100	100	100

For each of the eight *Candida* species analysed, precision (positive predictive value), sensitivity (true positive rate), and F1 scores (harmonic mean of precision and sensitivity values) are given. Values are taken from Random Forest models built using restricted mass range of 600 to 1000 *m/z*, a mass bin of 0.1 Da, and 400 decision making trees.
