# Supplementary material for: Rapid Evaporative Ionisation Mass Spectrometry (REIMS) Provides Accurate Direct from Culture Species Identification within the Genus Candida
Source: Sci Rep. 2016 Nov 14;6:36788. doi: 10.1038/srep36788 (PMC5107957; doi:10.1038/srep36788)

# Supporting Information

**Rapid Evaporative Ionisation Mass Spectrometry (REIMS) Provides Accurate Direct from Culture Species Identification within the Genus *Candida***

Simon JS Cameron (1)†; Frances Bolt (1)†; Alvaro Perdones-Montero (1); Tony Rickards (1,2); Kate Hardiman (1); Alireza Abdolrasouli (1,2,3); Adam Burke (1); Zsolt Bodai (1); Tamas Karancsi (4); Daniel Simon (4); Richard Schaffer (4); Monica Rebec (2); Julia Balog (4); Zoltan Takáts (1)*.

(1) Section of Computational and Systems Medicine, Department of Surgery and Cancer, Imperial College London, London, SW7 2AZ, United Kingdom; (2) Department of Microbiology, Imperial College Healthcare NHS Trust, Charing Cross Hospital, London, W6 8RF, United Kingdom; (3) Fungal Pathogens Immunobiology Laboratory, National Heart and Lung Institute, Imperial College London, SW7 2AZ, United Kingdom; (4) Waters Research Centre, 7 Zahony Street, Budapest, 1031, Hungary

† Joint first authors

* Communicating author: Prof. Zoltan Takáts. Email: z.takats@imperial.ac.uk

*This supporting material is provided to give more information on the experimental methodology employed in this study and to include additional experimental data referred to in the primary manuscript.*

**Table S1. Isolate Total and Culture Conditions for *Candida* Species Isolates Analysed**

The species name, abbreviation used in main manuscript body, culture conditions, including media type, temperature and length of incubation, and isolate total are given. CBA = Columbia Blood Agar.

| Species | Species Abbreviation | Culture Media | Temperature (°C) | Length (hrs) | Isolate Total | Conflicting Identification |
|---|---|---|---|---|---|---|
| *C. albicans* | CALB | CBA | 30 +/- 2 | 48 | 49 | 0 |
| *C. glabrata* | CGLA | CBA | 30 +/- 2 | 48 | 28 | 0 |
| *C. guilliermondii* | CGUI | CBA | 30 +/- 2 | 48 | 6 | 0 |
| *C. inconspicua* | CINC | CBA | 30 +/- 2 | 48 | 5 | 1 |
| *C. krusei* | CKRU | CBA | 30 +/- 2 | 48 | 12 | 2 |
| *C. lusitaniae* | CLUS | CBA | 30 +/- 2 | 48 | 6 | 2 |
| *C. parapsilosis* | CPAR | CBA | 30 +/- 2 | 48 | 35 | 1 |
| *C. tropicalis* | CTRO | CBA | 30 +/- 2 | 48 | 20 | 2 |

**Table S2. Operational Parameters of Xevo G2-XS Q-ToF Mass Spectrometer**

The operational conditions for the Xevo G2-XS Q-ToF instrument are given here.

| Parameter | Setting |
|---|---|
| Scan Time | 1000 ms |
| Scan Mode | Sensitive |
| Mass Analyser | Time of Flight |
| Ionisation Mode | Negative |
| Mass Range | 50 to 2500 |
| Sampling Cone | 80 V |
| Source Offset | 50 V |
| Source Temperature | 100 °C |

**Table S3. Tentative Lipid Identifications for Significant Features from Random Forest and ANOVA Analysis**

The top ten mass bins (0.1 Da) from Random Forest (RF) modelling and the highest ranking ANOVA mass bin (0.1 Da) for each *Candida* species are shown with tentative identifications using the LIPID MAPS database for (a) high-throughput REIMS analysis and (b) handheld bipolar REIMS analysis. The (1) mass bin from modelling or ANOVA is given, along with (2) the two decimal place mass of identified peak after interrogation of process spectra. The resulting (3) abbreviation of tentative lipid identification is given alongside the (4) matched mass of the compound to four decimal places, and the (5) LIPID MAPS Delta score value. The (6) molecular formula of the tentative lipid identification is given alongside the (7) negative ion. The (8) reference number indicates previously reported identifications of the tentative lipid within the *Candida* genus. The number relates to the numbered reference in the main manuscript.

**(a) High-Throughput REIMS Analysis**

| Source | m/z (1dp) (1) | m/z (2dp) (2) | Abbreviation (3) | Matched Mass (4) | Delta (5) | Formula (6) | Ion (7) | Reference (8) |
|---|---|---|---|---|---|---|---|---|
| **RF Feature 1** | 748.5 | 748.52 | PS(33:0) | 748.5134 | 0.0066 | $C_{39}H_{76}NO_{10}P$ | (M-H)- | |
| **RF Feature 2** | 746.5 | 746.53 | PS(O-34:1) | 746.5341 | 0.0041 | $C_{40}H_{77}NO_{9}P$ | (M-H)- | |
| | | | PS(P-34:0) | 746.5341 | 0.0041 | $C_{40}H_{77}NO_{9}P$ | (M-H)- | |
| **RF Feature 3** | 670.5 | 670.54 | HexCer(d32:1) | 670.5263 | 0.0137 | $C_{38}H_{72}NO_{8}$ | (M-H)- | |
| **RF Feature 4** | 747.5 | 747.52 | PG(34:1) | 747.5181 | 0.0019 | $C_{40}H_{76}O_{10}P$ | (M-H)- | 26 |
| **RF Feature 5** | 670.4 | 670.40 | PS(28:4) | 670.3725 | 0.0275 | $C_{34}H_{57}NO_{10}P$ | (M-H)- | |
| **RF Feature 6** | 745.5 | 670.45 | PC(28:3) | 670.4453 | 0.0047 | $C_{36}H_{65}NO_{8}P$ | (M-H)- | |
| **RF Feature 7** | 643.5 | 643.48 | PE-Cer(d33:2) | 643.4820 | 0.0020 | $C_{35}H_{68}N_{2}O_{6}P$ | (M-H)- | |
| | | | SM(d30:2) | 643.4820 | 0.0020 | $C_{35}H_{68}N_{2}O_{6}P$ | (M-H)- | |
| **RF Feature 8** | 748.6 | 748.60 | LPS(34:0) | 748.5498 | 0.0502 | $C_{40}H_{79}NO_{9}P$ | (M-H)- | |
| | | | PS(O-34:0) | 748.5498 | 0.0502 | $C_{40}H_{79}NO_{9}P$ | (M-H)- | |
| **RF Feature 9** | 747.8 | 747.82 | DG(O-46:1) | 747.7236 | 0.0964 | $C_{49}H_{95}O_{4}$ | (M-H)- | |
| **RF Feature 10** | 695.4 | 695.46 | PA(36:4) | 695.4657 | 00.0057 | $C_{39}H_{68}O_{8}P$ | (M-H)- | |
| **CALB ANOVA** | 695.4 | 695.46 | PA(36:4) | 695.4657 | 0.0057 | $C_{39}H_{68}O_{8}P$ | (M-H)- | 26 |
| **CGLA ANOVA** | 846.6 | 846.56 | PS(P-42:6) | 846.5654 | 0.0054 | $C_{48}H_{81}NO_{9}P$ | (M-H)- | |
| **CGUI ANOVA** | 859.5 | 859.53 | PI(36:3) | 859.5342 | 0.0042 | $C_{45}H_{80}O_{13}P$ | (M-H)- | 26, 27 |
| **CINC ANOVA** | 816.7 | 816.65 | PC(38:0) | 816.6488 | 0.0012 | $C_{46}H_{91}NO_{8}P$ | (M-H)- | |
| | | | PE(41:0) | 816.6488 | 0.0012 | $C_{46}H_{91}NO_{8}P$ | (M-H)- | |
| **CKRU ANOVA** | 738.7 | 738.70 | HexCer(d37:2) | 738.5889 | 0.1111 | $C_{43}H_{80}NO_{8}$ | (M-H)- | |
| **CLUS ANOVA** | 942.7 | 942.65 | SHexCer(d46:3) | 942.6709 | 0.0209 | $C_{52}H_{96}NO_{11}S$ | (M-H)- | |
| **CPAR ANOVA** | 838.5 | 838.55 | PS(40:4) | 838.5603 | 0.0103 | $C_{46}H_{81}NO_{10}P$ | (M-H)- | |
| **CTRO ANOVA** | 971.8 | 971.78 | PG(50:1) | 971.7685 | 0.0115 | $C_{56}H_{108}O_{10}P$ | [M-H]- | |

**(b) Handheld Bipolar REIMS Analysis**

| Source | m/z (1dp) (1) | m/z (2dp) (2) | Abbreviation (3) | Matched Mass (4) | Delta (5) | Formula (6) | Ion (7) | Reference (8) |
|---|---|---|---|---|---|---|---|---|
| **RF Feature 1** | 746.5 | 746.50 | PS(33:1) | 746.4977 | 0.0023 | $C_{39}H_{73}NO_{10}P$ | (M-H)- | |
| **RF Feature 2** | 747.5 | 747.51 | PG(34:1) | 747.5181 | 0.0019 | $C_{40}H_{76}O_{10}P$ | (M-H)- | |
| **RF Feature 3** | 745.5 | 745.50 | PG(34:2) | 745.5025 | 0.0025 | $C_{40}H_{74}O_{10}P$ | (M-H)- | 26 |
| **RF Feature 4** | 748.9 | 748.90 | Cer(d49:0) | 748.7552 | 0.1448 | $C_{49}H_{98}NO_3$ | (M-H)- | |
| **RF Feature 5** | 745.9 | 745.89 | WE(51:0) | 745.7807 | 0.0793 | $C_{51}H_{101}O_2$ | (M-H)- | |
| **RF Feature 6** | 836.5 | 836.53 | PE(44:11) | 836.5236 | 0.0064 | $C_{49}H_{75}NO_8P$ | (M-H)- | |
| **RF Feature 7** | 748.6 | 748.61 | LPS(34:0) | 748.5498 | 0.0602 | $C_{40}H_{79}NO_9P$ | (M-H)- | |
| | | | PS(O-34:0) | 748.5498 | 0.0602 | $C_{40}H_{79}NO_9P$ | (M-H)- | |
| **RF Feature 8** | 817.5 | 817.52 | PI(O-34:3) | 817.5236 | 0.0036 | $C_{43}H_{78}O_{12}P$ | (M-H)- | |
| | | | PI(P-34:2) | 817.5236 | 0.0036 | $C_{43}H_{78}O_{12}P$ | (M-H)- | |
| **RF Feature 9** | 749.5 | 749.52 | MGDG(34:4) | 749.5209 | 0.0009 | $C_{43}H_{73}O_{10}$ | (M-H)- | |
| **RF Feature 10** | 836.6 | 836.53 | PE(44:11) | 836.5236 | 0.0064 | $C_{49}H_{75}NO_8P$ | (M-H)- | |
| **CALB ANOVA** | 747.5 | 747.52 | PG(34:1) | 747.5181 | 0.0019 | $C_{40}H_{76}O_{10}P$ | (M-H)- | 26 |
| **CGLA ANOVA** | 846.6 | 846.56 | LacCer(t32:2) | 846.5584 | 0.0016 | $C_{44}H_{80}NO_{14}$ | (M-H)- | |
| **CGUI ANOVA** | 859.5 | 859.53 | PI(36:3) | 859.5342 | 0.0042 | $C_{45}H_{80}O_{13}P$ | (M-H)- | 26, 27 |
| **CINC ANOVA** | 682.5 | 682.53 | HexCer(d33:2) | 682.5263 | 0.0037 | $C_{39}H_{72}NO_8$ | (M-H)- | |
| **CKRU ANOVA** | 613.5 | 613.46 | DG(36:5) | 613.4837 | 0.0237 | $C_{39}H_{65}O_5$ | (M-H)- | |
| **CLUS ANOVA** | 940.6 | 940.64 | MIPC(m35:0) | 940.6132 | 0.0268 | $C_{47}H_{91}NO_{15}P$ | (M-H)- | |
| **CPAR ANOVA** | 849.6 | 849.55 | PI(35:1) | 849.5498 | 0.0002 | $C_{44}H_{82}O_{13}P$ | (M-H)- | 26, 27 |
| **CTRO ANOVA** | 949.6 | 949.55 | PIP(O-38:5) | 949.5213 | 0.0287 | $C_{47}H_{83}O_{15}P_2$ | (M-H)- | |

**Figure S1. Mean Mass Spectra of *C. albicans* Isolates using Handheld Bipolar Probe and High-Throughput REIMS Approaches**

The mean mass spectra for all *C. albicans* isolates are given, following background subtraction, mass drift correction, and removal of lock mass compound (leu-enkaphaline), and its isotopic peak, at mass bins 554 and 555 respectively, using a mass bin of 1 Da.

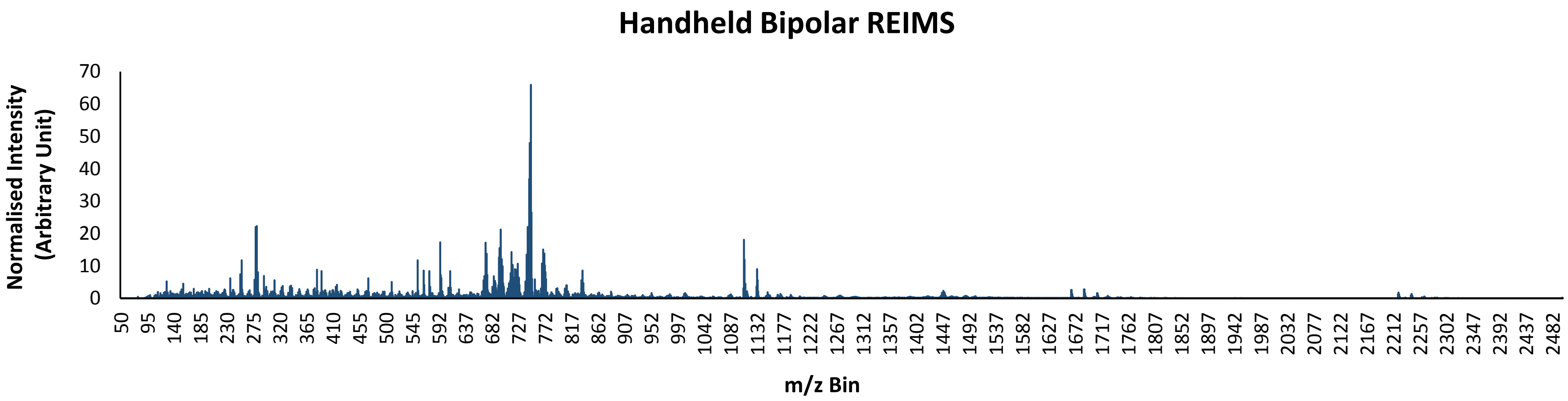


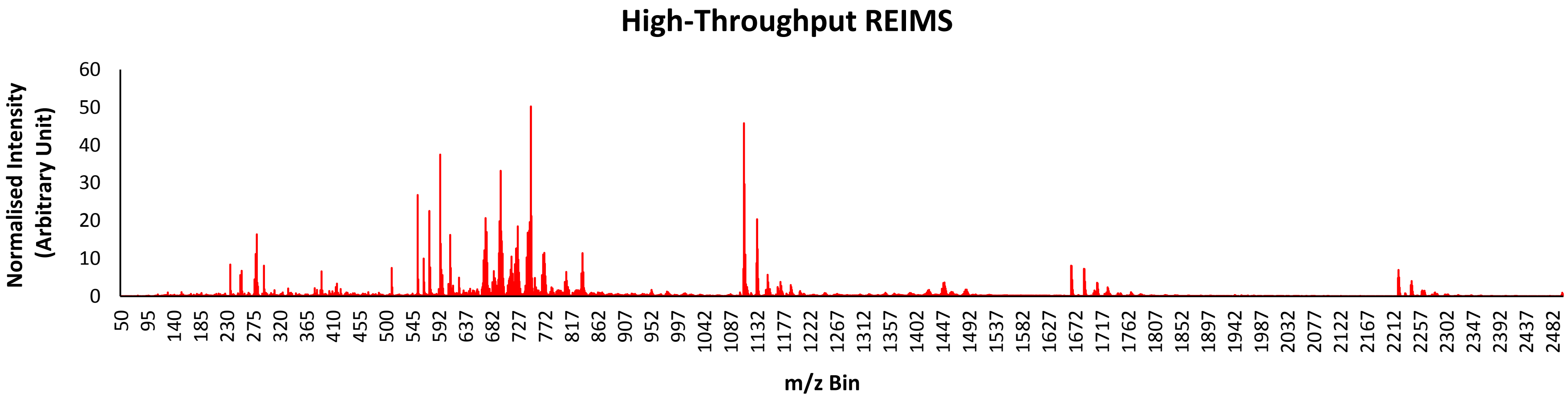

**Figure S2. Mean Mass Spectra of *C. glabrata* Isolate using Handheld Bipolar Probe and High-Throughput REIMS Approaches**

The mean mass spectra for all *C. glabrata* isolates are given, following background subtraction, mass drift correction, and removal of lock mass compound (leu-enkaphaline), and its isotopic peak, at mass bins 554 and 555 respectively, using a mass bin of 1 Da.

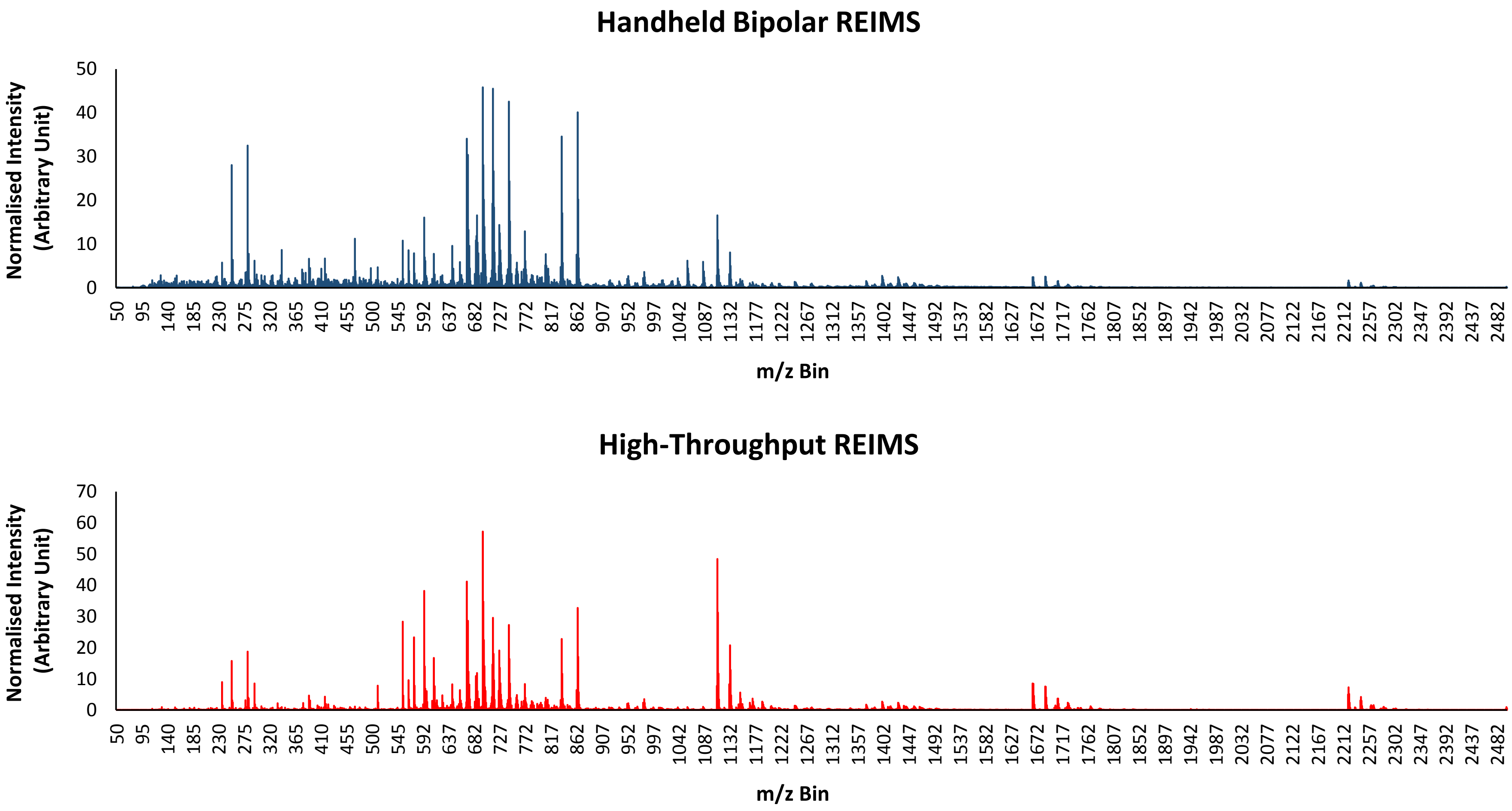

**Figure S3. Mean Mass Spectra of *C. guilliermondii* Isolate using Handheld Bipolar Probe and High-Throughput REIMS Approaches**

The mean mass spectra for all *C. guilliermondii* isolates are given, following background subtraction, mass drift correction, and removal of lock mass compound (leu-enkaphaline), and its isotopic peak, at mass bins 554 and 555 respectively, using a mass bin of 1 Da.

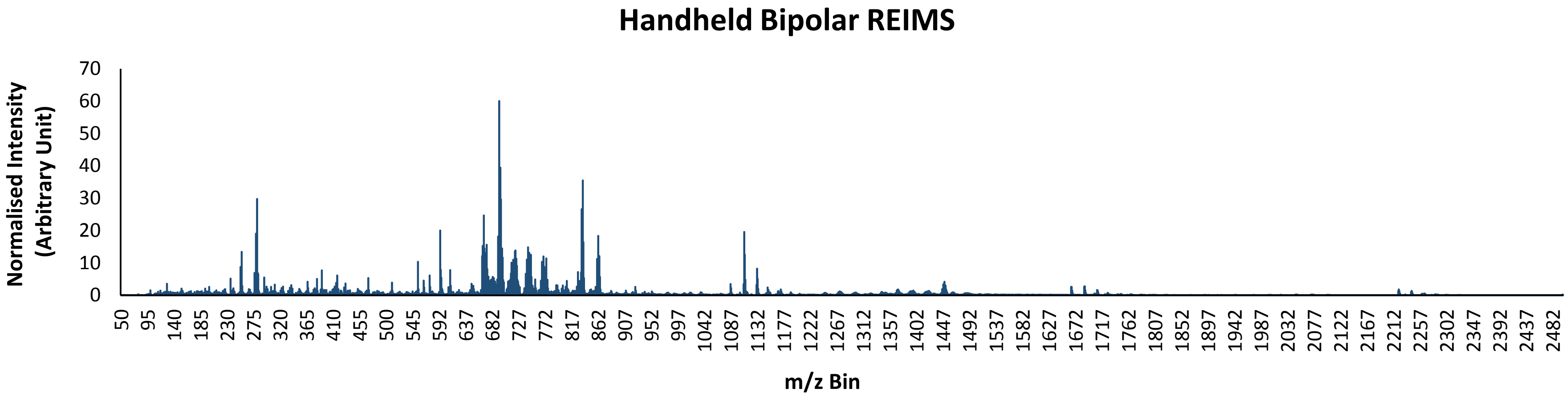


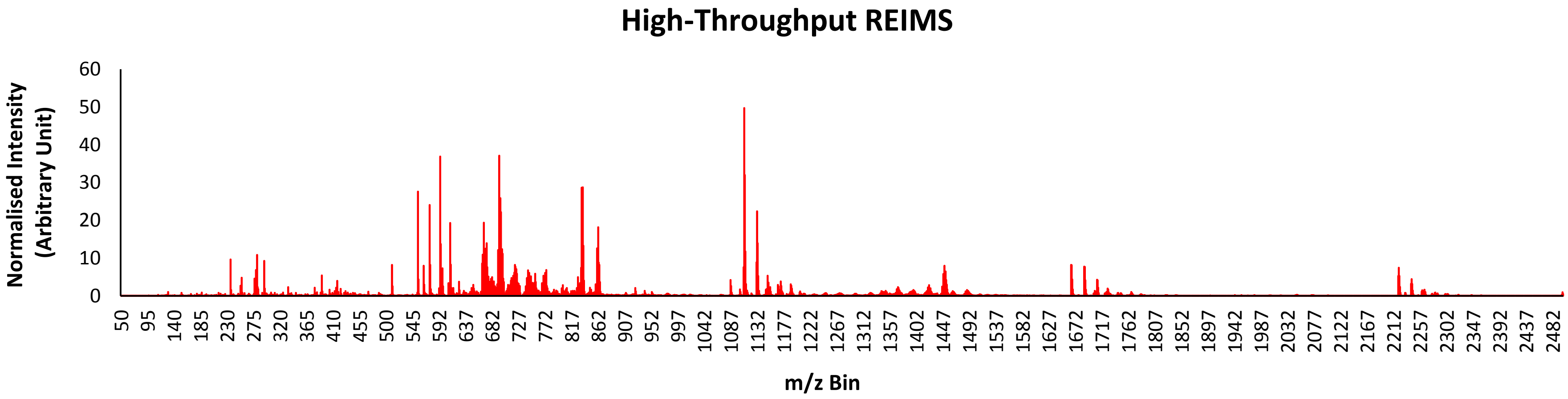

**Figure S4. Mean Mass Spectra of *C. inconspicua* Isolate using Handheld Bipolar Probe and High-Throughput REIMS Approaches**

The mean mass spectra for all *C. inconspicua* isolates are given, following background subtraction, mass drift correction, and removal of lock mass compound (leu-enkaphaline), and its isotopic peak, at mass bins 554 and 555 respectively, using a mass bin of 1 Da.

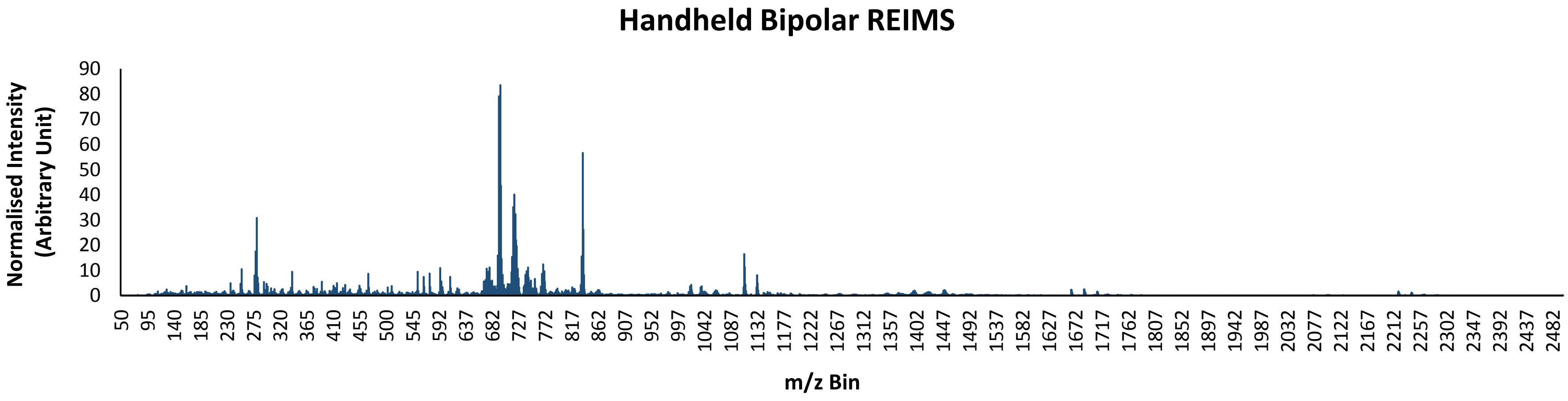


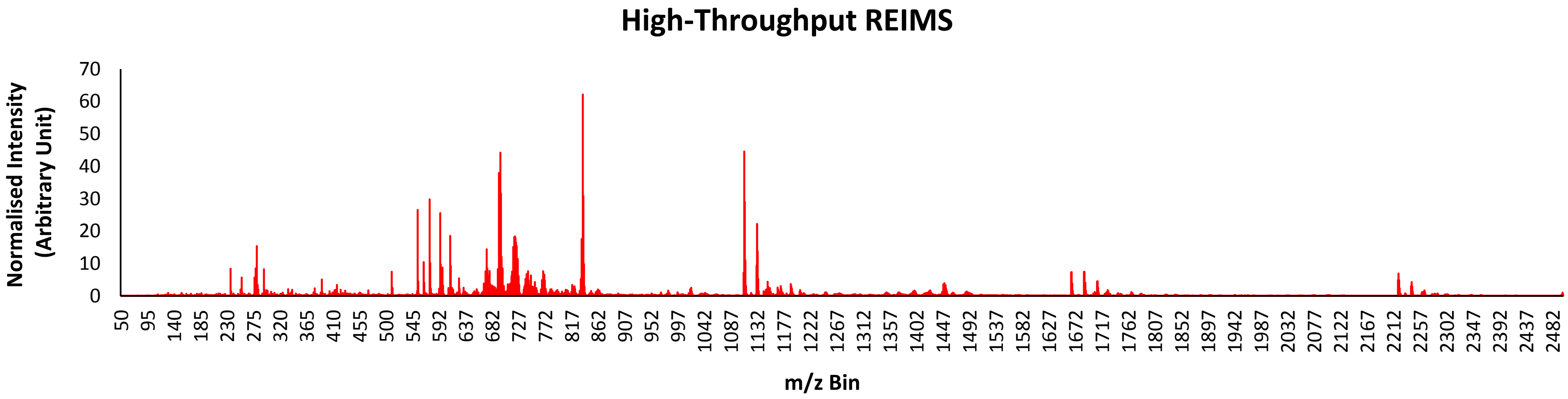

**Figure S5. Mean Mass Spectra of *C. krusei* Isolate using Handheld Bipolar Probe and High-Throughput REIMS Approaches**

The mean mass spectra for all *C. krusei* isolates are given, following background subtraction, mass drift correction, and removal of lock mass compound (leu-enkaphaline), and its isotopic peak, at mass bins 554 and 555 respectively, using a mass bin of 1 Da.

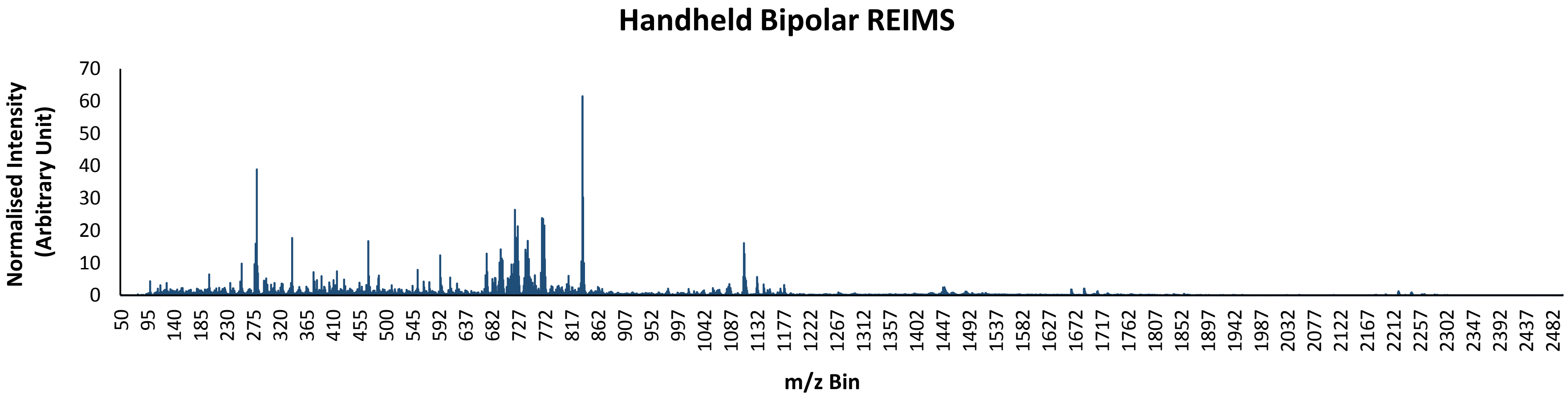


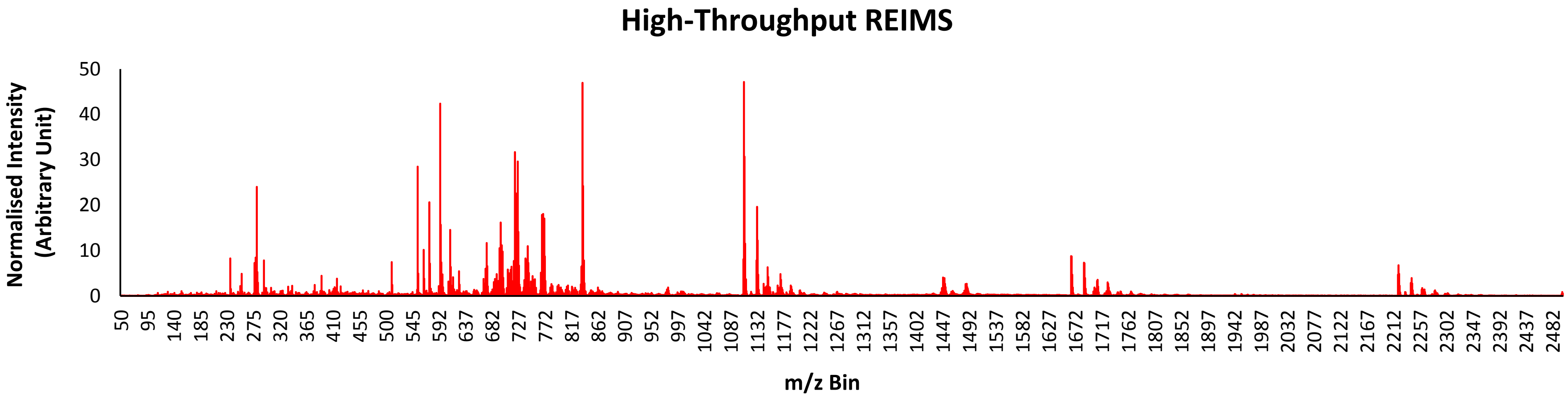

**Figure S6. Mean Mass Spectra of *C. lusitaniae* Isolate using Handheld Bipolar Probe and High-Throughput REIMS Approaches**

The mean mass spectra for all *C. lusitaniae* isolates are given, following background subtraction, mass drift correction, and removal of lock mass compound (leu-enkaphaline), and its isotopic peak, at mass bins 554 and 555 respectively, using a mass bin of 1 Da.

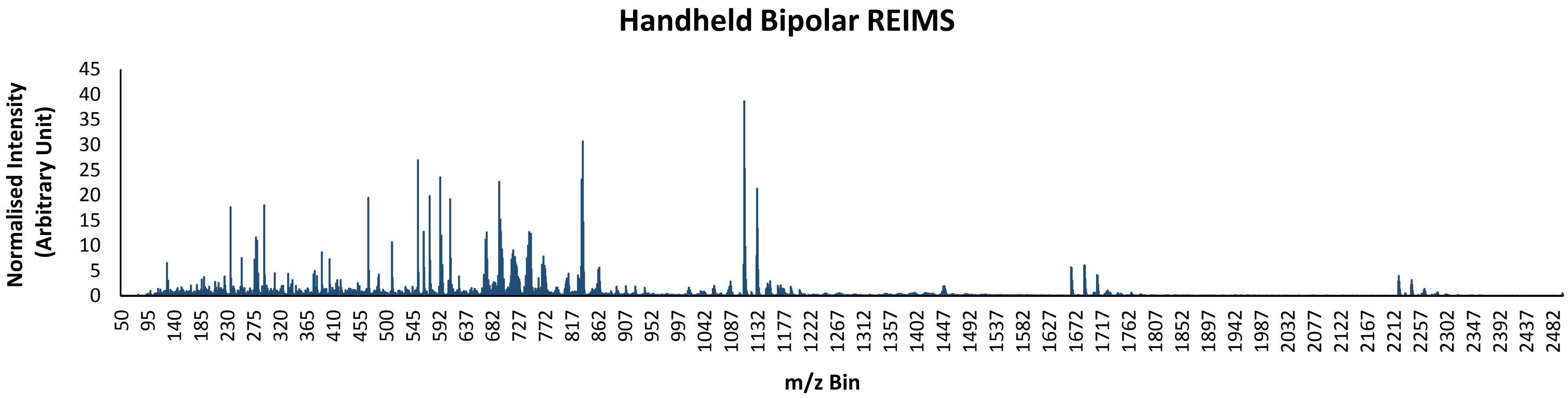


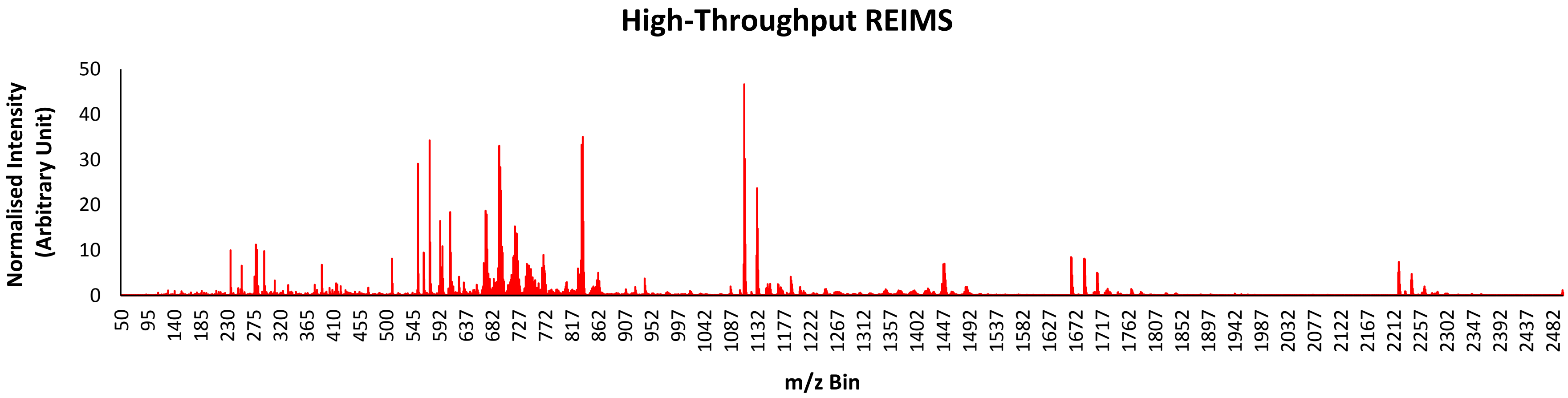

**Figure S7. Mean Mass Spectra of *C. parapsilosis* Isolate using Handheld Bipolar Probe and High-Throughput REIMS Approaches**

The mean mass spectra for all *C. parapsilosis* isolates are given, following background subtraction, mass drift correction, and removal of lock mass compound (leu-enkaphaline), and its isotopic peak, at mass bins 554 and 555 respectively, using a mass bin of 1 Da.

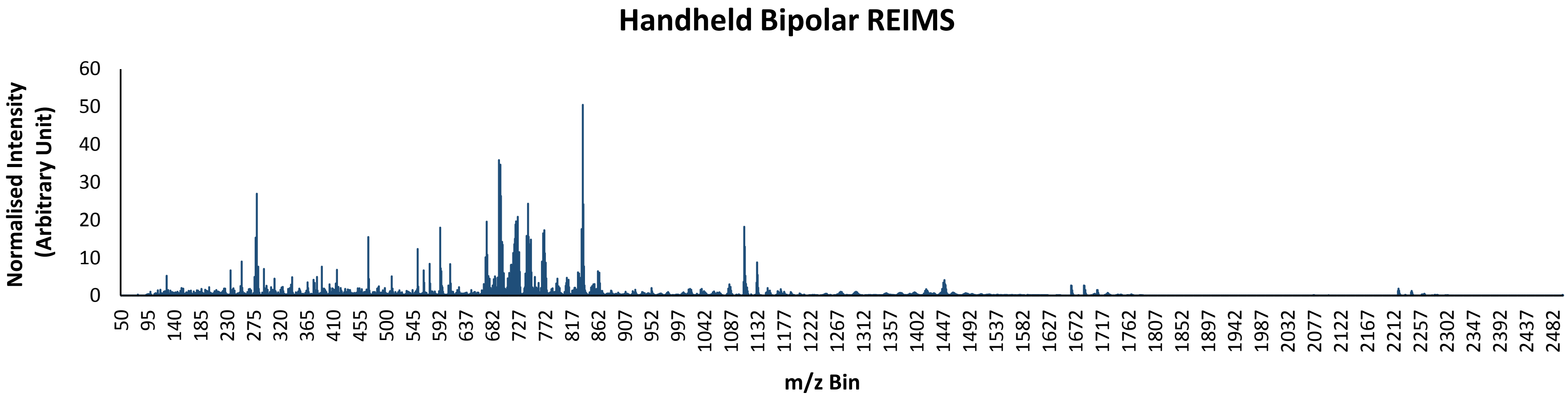


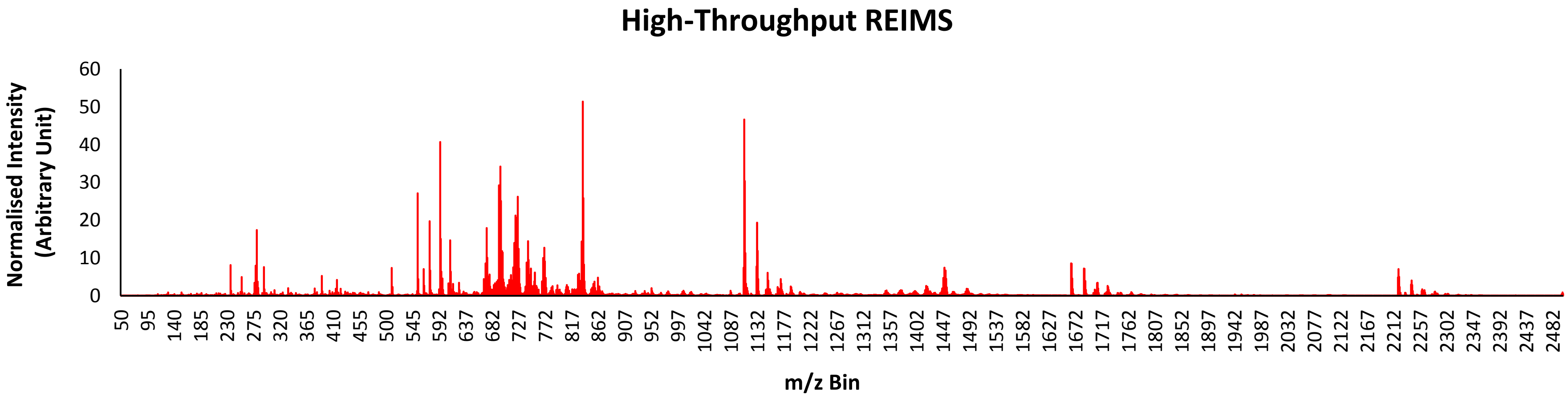

**Figure S8. Mean Mass Spectra of *C. tropicalis* Isolate using Handheld Bipolar Probe and High-Throughput REIMS Approaches**

The mean mass spectra for all *C. tropicalis* isolates are given, following background subtraction, mass drift correction, and removal of lock mass compound (leu-enkaphaline), and its isotopic peak, at mass bins 554 and 555 respectively, using a mass bin of 1 Da.

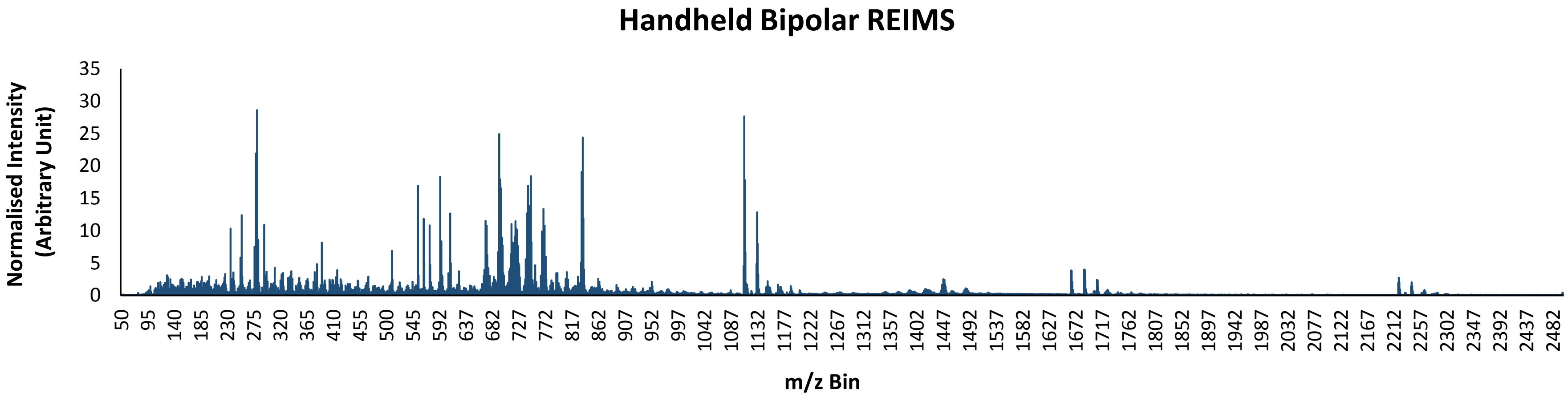


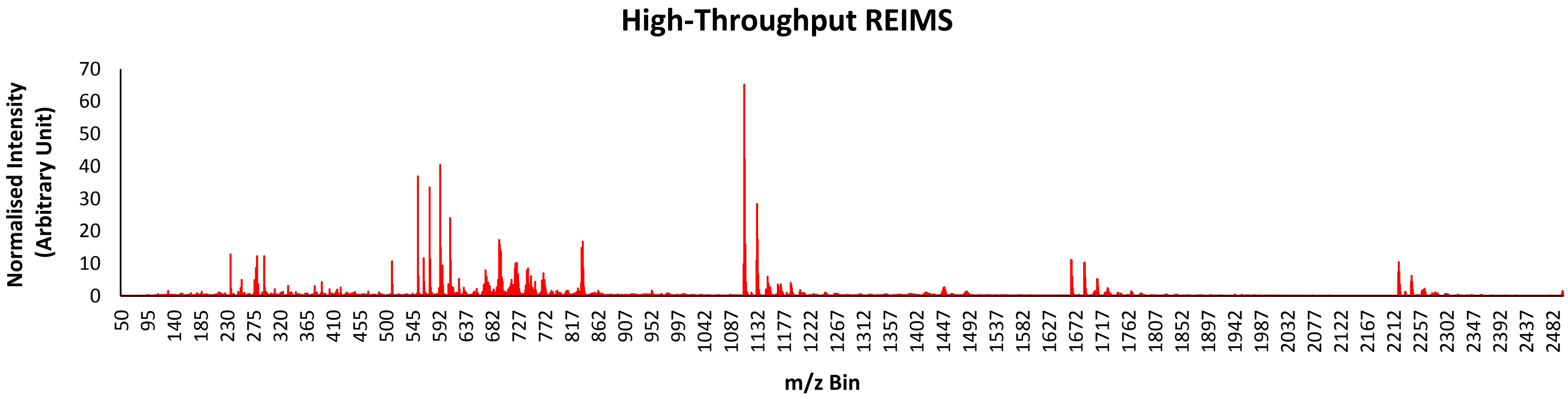

**Figure S9. Random Forest Feature Selection from 50 to 2500 *m/z* Range**

For both REIMS approaches, the top 250 mass bins, at 0.1 Da, were selected based on their importance in Random Forest classification models. The total (blue bars) number of features within each mass bin range, in divisions of 100, are shown along with the sum importance (orange) line for both (a) handheld bipolar REIMS, and (b) high-throughput REIMS.

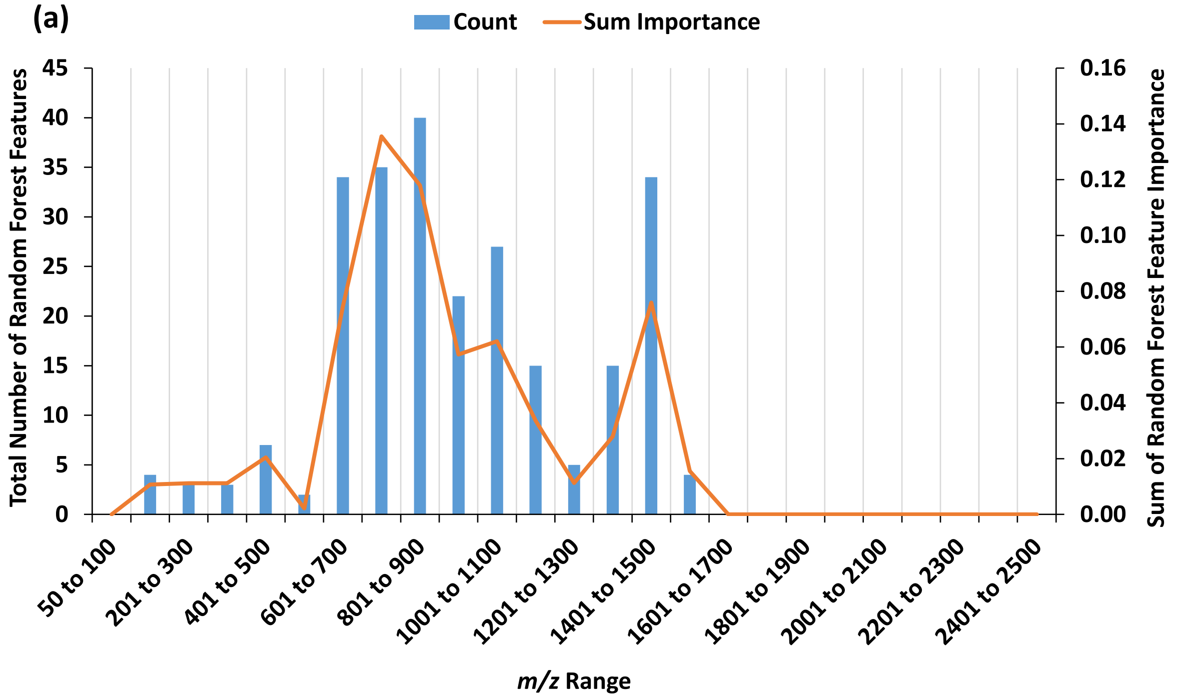


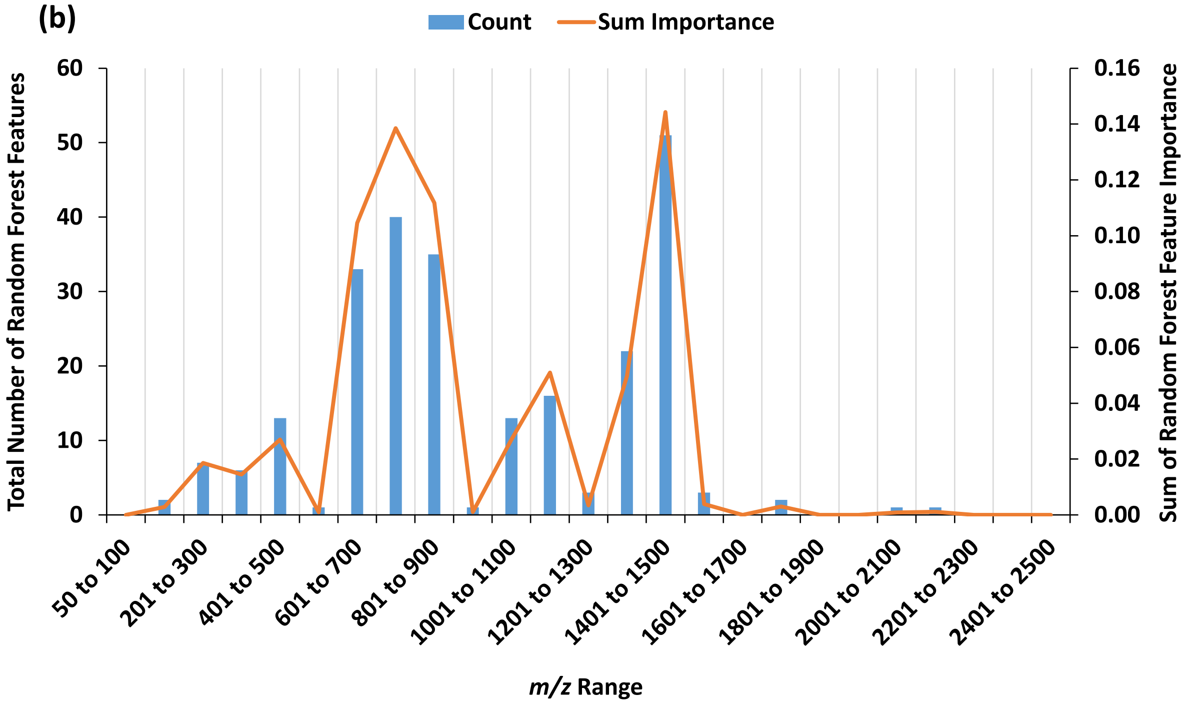

**Figure S10. Effect of Mass Bin Size on Species Classification Accuracy**

For both REIMS approaches, the species classification accuracy of mass bin sizes of 0.01 Da, 0.05 Da, 0.1 Da, 0.5 Da, and 1 Da, within the restricted mass range of 600 to 1000 *m/z*, was tested. Random Forest models constructed used 400 decision making trees.

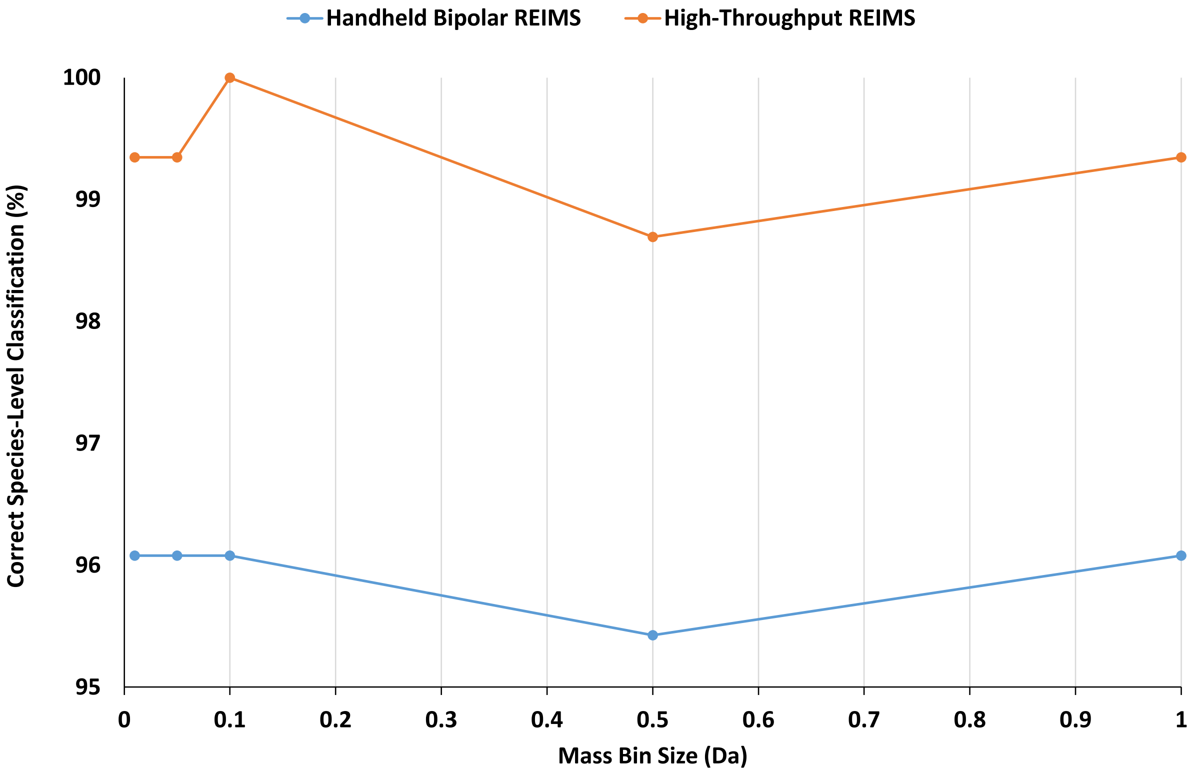

**Figure S11. Number of Decision Making Trees Required for Accuracy Plateau to be Reached**

A range of decision making tree numbers were tested for Random Forest models, ranging from 10 to 50 in increments of 10; 100 to 400 in increments of 100; and 500 to 2000 in increments of 500. For all numbers of decision making trees, high-throughput REIMS outperformed handheld bipolar REIMS with regard to species level classification accuracy.

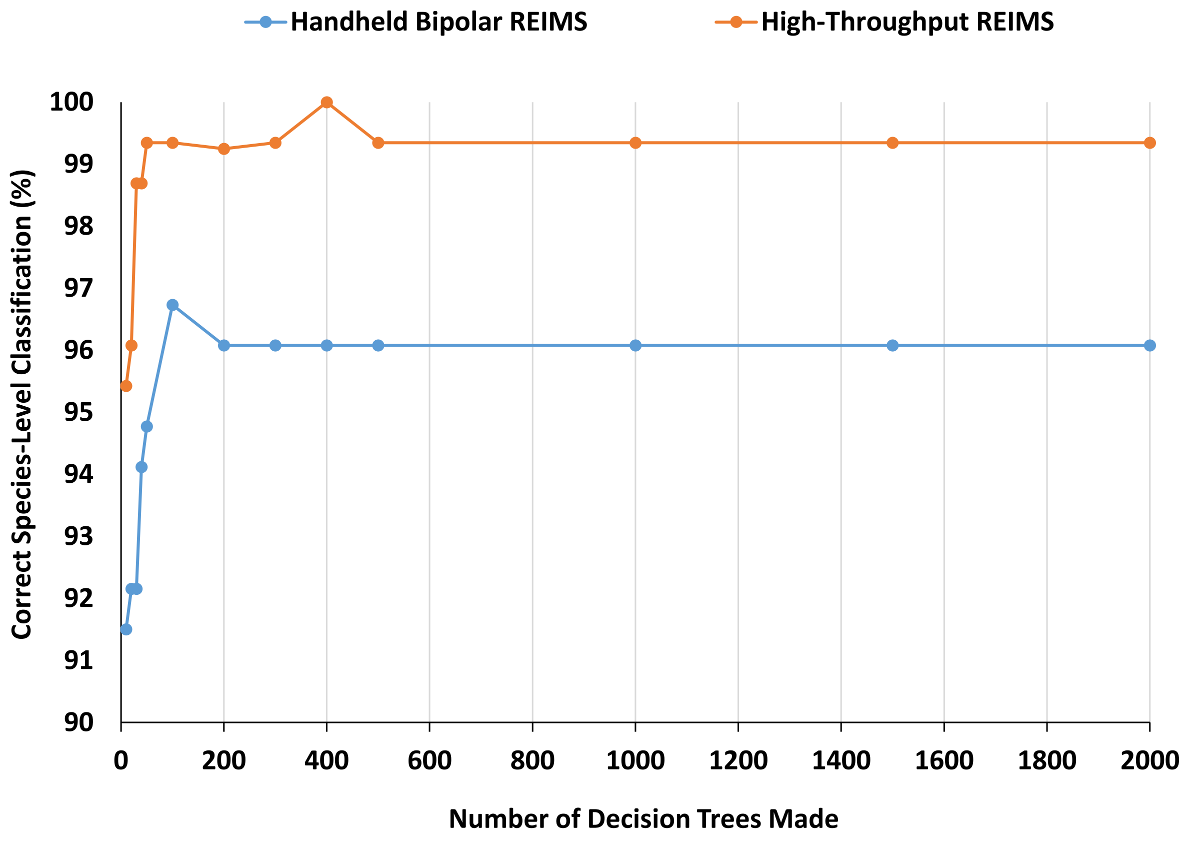

Supplement: Supplementary Information [file srep36788-s1.pdf]
